# Assistive artificial intelligence for ultrasound image interpretation in regional anaesthesia: an external validation study

**DOI:** 10.1016/j.bja.2022.06.031

**Published:** 2022-08-18

**Authors:** James S. Bowness, David Burckett-St Laurent, Nadia Hernandez, Pearse A. Keane, Clara Lobo, Steve Margetts, Eleni Moka, Amit Pawa, Meg Rosenblatt, Nick Sleep, Alasdair Taylor, Glenn Woodworth, Asta Vasalauskaite, J. Alison Noble, Helen Higham

**Affiliations:** 1 Oxford Simulation, Teaching and Research Centre, University of Oxford, Oxford, UK; 2 Department of Anaesthesia, Aneurin Bevan University Health Board, Newport, UK; 3 Department of Anaesthesia, Royal Cornwall Hospitals NHS Trust, Truro, UK; 4 Department of Anesthesiology, Memorial Hermann Hospital, Texas Medical Centre, Houston, TX, USA; 5 Institute of Ophthalmology, Faculty of Brain Sciences, University College London, London, UK; 6 National Institute for Health and Care Research Biomedical Research Centre, Moorfields Eye Hospital NHS Foundation Trust, London, UK; 7 Anesthesiology Institute, Cleveland Clinic Abu Dhabi, Abu Dhabi, United Arab Emirates; 8 Intelligent Ultrasound, Cardiff, UK; 9 Anaesthesiology Department, Creta InterClinic Hospital, Hellenic Healthcare Group, Heraklion, Crete, Greece; 10 Department of Anaesthesia, Guy's and St Thomas' Hospitals NHS Trust, London, UK; 11 Faculty of Life Sciences and Medicine, King's College London, London, UK; 12 Department of Anesthesiology, Perioperative and Pain Medicine, Mount Sinai Morningside and West Hospitals, New York, NY, USA; 13 Department of Anaesthesia, NHS Tayside, Dundee, UK; 14 Department of Anesthesiology and Perioperative Medicine, Oregon Health & Science University, Portland, OR, USA; 15 Institute of Biomedical Engineering, University of Oxford, Oxford, UK; 16 Department of Anaesthesia, Oxford University Hospitals NHS Foundation Trust, Oxford, UK

**Keywords:** anatomy, artificial intelligence, machine learning, regional anaesthesia, translational AI, ultrasonography, ultrasound

## Abstract

**Background:**

Ultrasonound is used to identify anatomical structures during regional anaesthesia and to guide needle insertion and injection of local anaesthetic. ScanNav Anatomy Peripheral Nerve Block (Intelligent Ultrasound, Cardiff, UK) is an artificial intelligence-based device that produces a colour overlay on real-time B-mode ultrasound to highlight anatomical structures of interest. We evaluated the accuracy of the artificial-intelligence colour overlay and its perceived influence on risk of adverse events or block failure.

**Methods:**

Ultrasound-guided regional anaesthesia experts acquired 720 videos from 40 volunteers (across nine anatomical regions) without using the device. The artificial-intelligence colour overlay was subsequently applied. Three more experts independently reviewed each video (with the original unmodified video) to assess accuracy of the colour overlay in relation to key anatomical structures (true positive/negative and false positive/negative) and the potential for highlighting to modify perceived risk of adverse events (needle trauma to nerves, arteries, pleura, and peritoneum) or block failure.

**Results:**

The artificial-intelligence models identified the structure of interest in 93.5% of cases (1519/1624), with a false-negative rate of 3.0% (48/1624) and a false-positive rate of 3.5% (57/1624). Highlighting was judged to reduce the risk of unwanted needle trauma to nerves, arteries, pleura, and peritoneum in 62.9–86.4% of cases (302/480 to 345/400), and to increase the risk in 0.0–1.7% (0/160 to 8/480). Risk of block failure was reported to be reduced in 81.3% of scans (585/720) and to be increased in 1.8% (13/720).

**Conclusions:**

Artificial intelligence-based devices can potentially aid image acquisition and interpretation in ultrasound-guided regional anaesthesia. Further studies are necessary to demonstrate their effectiveness in supporting training and clinical practice.

**Clinical trial registration:**

NCT04906018.

**Editor's key points**

• Ultrasound-guided regional anaesthesia facilitates precision, safety, and effectiveness of peripheral nerve block, but it is technically challenging without advanced training.
• The use of ScanNav™ (Intelligent Ultrasound, Cardiff, UK), an artificial intelligence-based device that produces a colour overlay on real-time ultrasound images to highlight anatomical structures of interest, was evaluated.
• Experts reviewed 720 ultrasound videos, with and without ScanNav^TM^ highlighting, to assess accuracy and perceived effect on regional anaesthesia safety and efficacy.
• The device showed high true-positive/true-negative and low false-positive/false-positive rates in identifying key anatomical structures for the performance of nine peripheral nerve blocks.
• Further studies are necessary to demonstrate its effectiveness in supporting training and clinical practice.

The use of ultrasound as image guidance for regional anaesthesia was first described in 1989^1^ and is now the predominant technique used to guide needle insertion and local anaesthetic deposition.^2^ Ultrasound-guided regional anaesthesia (UGRA) can be used to avoid risks associated with general anaesthesia,^3^ enhance operating theatre efficiency, and reduce hospital length of stay.^4^ Evidence also supports a role in improving outcomes after surgery^4^^,^^5^ and in mitigating the need for systemic analgesia with dangerous side-effects, such as opioids.^2^^,^^4^

However, patient access to UGRA can be limited by the availability of a specialist with prerequisite knowledge and skills.^6^ Fundamental skills are the acquisition and interpretation of optimal ultrasound images, which involves identification of key sono-anatomical structures.^7^ Assistive artificial intelligence (AI) technology could play a role in the future practice of UGRA through supporting ultrasound scanning.^8^^,^^9^ ScanNav Anatomy Peripheral Nerve Block (Intelligent Ultrasound, Cardiff, UK) uses deep learning based on the U-Net architecture^10^ to produce a colour overlay on real-time B-mode ultrasound and highlight structures of interest in UGRA (Fig 1; Supplementary files A–E). The AI models in this system have been trained on more than 800,000 ultrasound images.^11^ Training data are presented to the algorithm as paired unmodified ultrasound image and labelled colour overlay (highlighting the relevant structures on that image). Over time, the algorithm learns to make associations between the labelled region and data in the image. When deployed, it is thus able to make predictions on data in new images and provide a colour overlay on real-time ultrasound. (Further information on the training data is available in Supplementary file F.) It is envisaged that a real-time colour overlay will draw attentional gaze of the operator to the key anatomical structures. Previous work supports the concept that it can aid in acquisition of the correct ultrasound view and correct identification of structures of interest on that view.^12^

**Fig 1:**
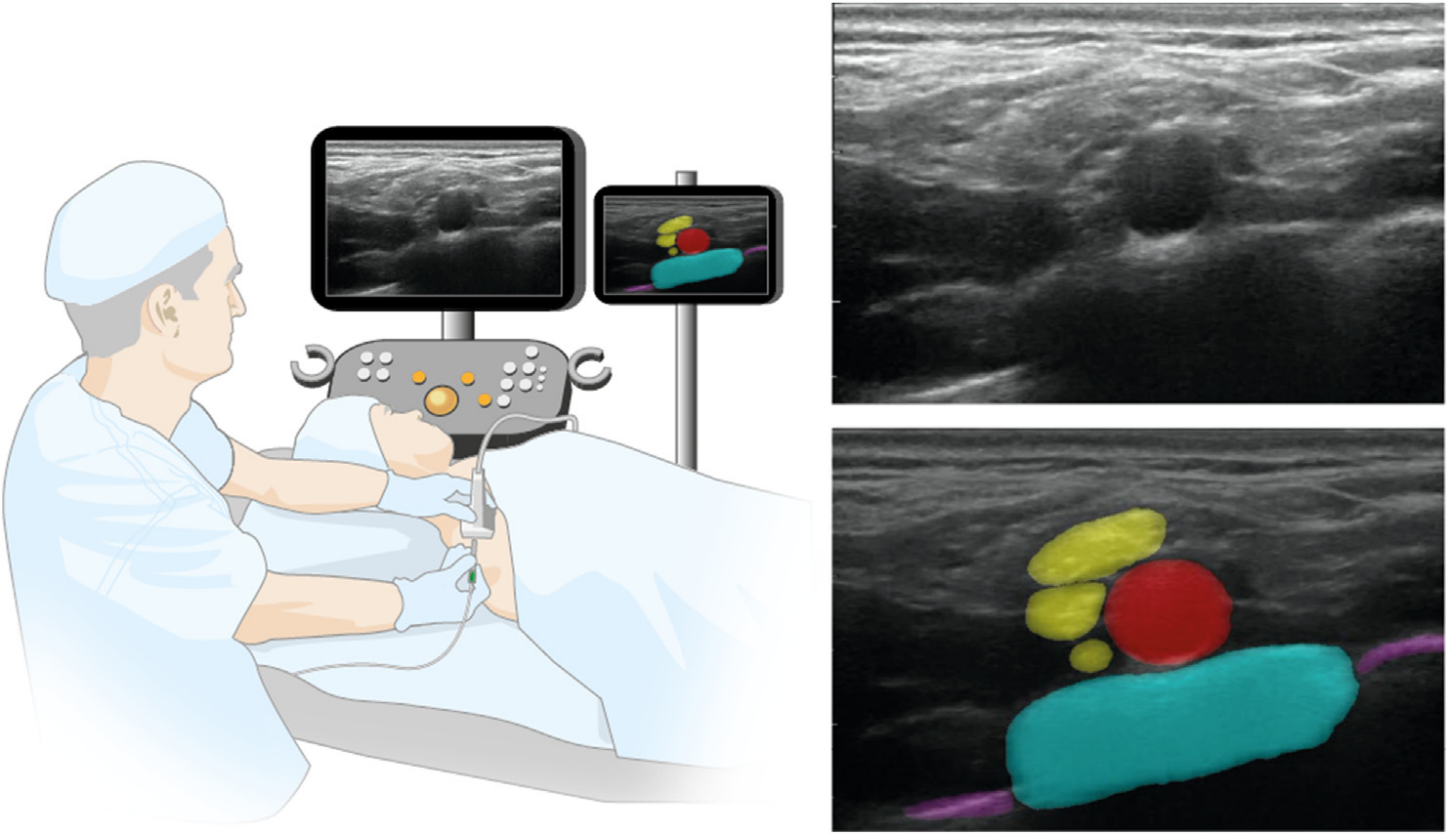
Example of the colour overlay produced by ScanNav when scanning during a supraclavicular-level brachial plexus block. Blue, first rib; purple, pleura; red, subclavian artery; and yellow, supraclavicular-level brachial plexus nerves (trunks/divisions).

In this prospective external validation study, experts in UGRA acquired ultrasound scans (without use of ScanNav), and further experts evaluated performance of the AI models. Accuracy of the colour overlay was assessed in relation to key anatomical structures. The perceived potential for highlighting to modify the risk of adverse events (i.e. risk of needle trauma to nerves, arteries, pleura, and peritoneum) and block failure was also evaluated.

## Methods

Ethical approval for the collection of ultrasonography scans from healthy volunteers was granted by the Oregon Health & Science University (OHSU) Institutional Review Board (STUDY00022920). The study was registered with ClinicalTrials.gov (NCT04906018).

### Ultrasonography scan collection

**Fig 2:**
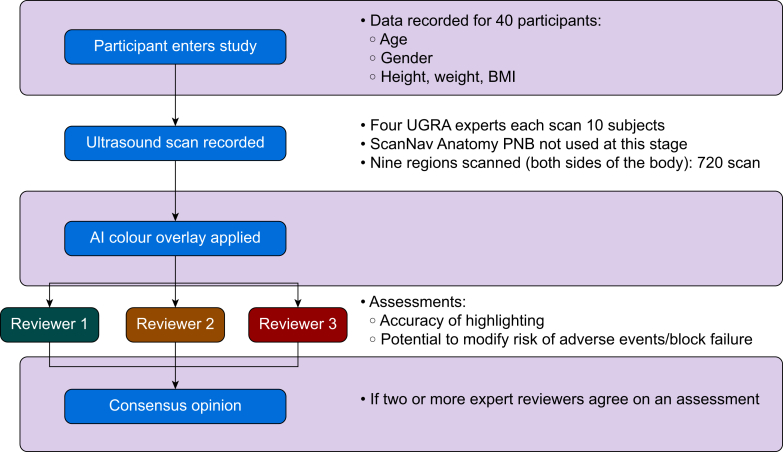
Summary of study workflow. AI, artificial intelligence; PNB, peripheral nerve block; UGRA, ultrasound-guided regional anaesthesia.

The process of scan acquisition and review is summarised in Fig 2. Four UGRA experts were recruited from the anaesthesia faculty at OHSU after providing written informed consent. All were board-certified attending anaesthesiologists who had completed advanced training in UGRA (fellowship or equivalent) and regularly use these techniques in their clinical practice.

Forty healthy adult subjects were recruited for scanning after providing written informed consent. Exclusion criteria were age <18 yr and known pathology affecting the areas scanned. Scanning was performed using the SonoSite X-Porte (HFL50xp and L38xp linear probes or C60xp curvilinear probe) and PX (L15–5 and L12-3 linear probes or C5-1 curvilinear probe) ultrasound machines (FUJIFILM SonoSite, Bothell, WA, USA).

Each expert scanned 10 subjects (bilaterally) without ScanNav over anatomical regions relevant to nine specific peripheral nerve blocks (PNBs). Upper-limb block regions scanned included the interscalene-level brachial plexus, upper trunk of the brachial plexus, supraclavicular-level brachial plexus (SC), and the axillary-level brachial plexus (AxBP) blocks. Thoracoabdominal block regions included the erector spinae plane (ESP) and rectus sheath (RSB) blocks. Lower-limb block regions comprised the suprainguinal fascia iliaca, adductor canal/distal femoral triangle, and popliteal-level sciatic nerve blocks.

A total of 720 scans were performed. For each scan, the scanner stated when they had acquired what they felt to be the optimal view. This frame and the preceding 10 s of the scan were used for later review. Predictive colour overlay, derived by ScanNav^TM^, was subsequently applied to the ultrasound clips obtained in the acquisition protocol.

### Key anatomical structures and adverse events

Nerves, arteries, pleura, and peritoneum were considered as safety-critical structures (although the pleura, in the context of the ESP block, is not typically in view when the needle is inserted,^13^ and thus, risk of pneumothorax is low). Target structures for UGRA include peripheral nerves and fascial planes. Therefore, highlighting of the rectus sheath and fascia iliaca and the transverse process of thoracic vertebrae were assessed. The structures for each PNB are detailed in Table 1.

**Table 1:** Key anatomical structures for ultrasonography-guided regional anaesthesia safety and block success.

Peripheral nerve block region	Nerve	Artery	Serosal plane	Bone	Fascia
Interscalene-level brachial plexus block	C5 and C6 nerve roots				
Upper-trunk block	Upper trunk of brachial plexus				
Supraclavicular-level brachial plexus block	Trunks/divisions of brachial plexus	Subclavian artery	Pleura		
Axillary-level brachial plexus block	Musculocutaneous, median, ulnar, and radial nerves	Axillary artery			
Erector spinae plane block			Pleura	Transverse process	
Rectus sheath block			Peritoneum		Rectus sheath
Suprainguinal fascia iliaca block		Deep circumflex iliac artery			Fascia iliaca
Adductor canal block	Saphenous nerve	Femoral artery			
Popliteal-level sciatic nerve block	Sciatic nerve	Popliteal artery			

### Expert reviewer evaluation

Six additional UGRA experts were recruited for analysis of the highlighting on the recorded scans. Three were based in the USA (board-certified attending anaesthesiologists) and three in Europe (consultant anaesthetist or equivalent). All had completed advanced training in UGRA (fellowship or equivalent) and regularly use these techniques in their clinical practice.

Unmodified ultrasound scans and colour-highlighted scan pairs were presented to expert reviewers *via* an online platform. Videos in the pair played simultaneously with the expert reviewers at liberty to play/pause at their discretion and view multiple times. Scans were labelled with the subject age, sex, and BMI. Three expert reviewers assessed each scan independently: none knew the scans allocated to other expert reviewers or the outcome of their evaluation. A consensus view was taken for each assessment; in cases where no majority was reached, this was classified as ‘no consensus’.

For the relevant structures in each scan, reviewers were asked to appraise highlighting accuracy and associated adverse events through the following statements:

(i) The [colour] highlighting for the [anatomical structure]
(a) Correctly identifies the [anatomical structure] (true positive; TP)
(b) Is in the wrong location (false positive; FP)
(c) Is not present and the [anatomical structure] is not present (true negative; TN)
(d) Is not present but the [anatomical structure] is present (false negative; FN)
(ii) Regarding the risk of [specific adverse event], the highlighting seen in this clip
(a) Increases the risk of [specific adverse event]
(b) Does not change the risk of [specific adverse event]
(c) Reduces the risk of [specific adverse event]
(iii) Regarding the risk of block failure, the highlighting seen in this clip
(a) Increases the risk of block failure
(b) Does not change the risk of block failure
(c) Reduces the risk of block failure

### Statistical analysis

As this study used a clinical and subjective assessment of the AI models, descriptive statistics of both accuracy and efficacy (perceived influence on risk of adverse event or block failure) have been reported in a manner that reflects clinical use. As all structures for a block region can be present or absent on any single scan, the reported accuracy is presented for each PNB and overall. Accuracy was defined as the sum of the true-positive rate (TPr; TP/total structures) and true-negative rate (TNr; TN/total structures). Rates of false positive (FPr) and false negative (FNr) were similarly calculated but reported independently because of the clinical implications of discriminating between FP and FN.

## Results

Mean age of the scan subjects was 41.2 (min–max: 23–64; standard deviation [sd] 13.4) yr, and mean BMI was 28.9 (19.7–40.4; 6.1) kg m^−2^, with an equal male:female ratio.

### Accuracy

Table 2 shows a summary of the accuracy assessments made by the expert reviewers. Twenty-one key anatomical structures were considered across nine PNBs. Each PNB region was scanned 80 times; thus, a total of 1680 key anatomical structures were assessed, each one by three expert reviewers. A majority view of expert opinion was determined in 1624 structures (96.7%); no consensus was reached in 56 (3.3%).

**Table 2:** Perceived accuracy assessment by peripheral nerve block. FNr, false-negative rate; FPr, false-positive rate; TNr, true-negative rate; TPr, true-positive rate.

Peripheral nerve block	True positive		True negative		False positive		False negative		Accuracy (TPr+TNr)	Total structures
	TPr	Structures	TNr	Structures	FPr	Structures	FNr	Structures		
Interscalene-level brachial plexus block	0.908	139	0.033	5	0.013	2	0.046	7	0.941	153
Upper-trunk block	0.896	69	0.013	1	0.052	4	0.039	3	0.909	77
Supraclavicular-level brachial plexus block	0.958	226	0.025	6	0.008	2	0.008	2	0.983	236
Axillary-level brachial plexus block	0.951	366	0.026	10	0.003	1	0.021	8	0.977	385
Erector spinae plane block	0.638	97	0.250	38	0.000	0	0.112	17	0.888	152
Rectus sheath block	0.968	149	0.000	0	0.032	5	0.000	0	0.968	154
Suprainguinal fascia iliaca block	0.702	106	0.060	9	0.219	33	0.020	3	0.762	151
Adductor canal block	0.872	136	0.032	5	0.000	0	0.096	15	0.904	156
Popliteal-level sciatic nerve block	0.875	140	0.106	17	0.006	1	0.013	2	0.981	160
Average/total	0.879	1428	0.056	91	0.030	48	0.035	57	0.935	1624

Mean accuracy (TPr+TNr) was 93.5% (1519/1624; TPr 87.9% and TNr 5.6%; min–max accuracy: 76.2–98.3; sd 6.7; 95% confidence interval [CI]: 89.1–97.9). Rate of structure misidentification (FPr) was 3.0% (48/1624; 0–21.9; sd 6.6; 95% CI: 0.0–7.3) and non-identification of a structure (FNr) was 3.5% (57/1624; 0–11.2; sd 3.7; 95% CI: 1.1–5.9). Further detail for each block and structure is presented in Supplementary file F.

### Adverse events and block failure

**Fig 3:**
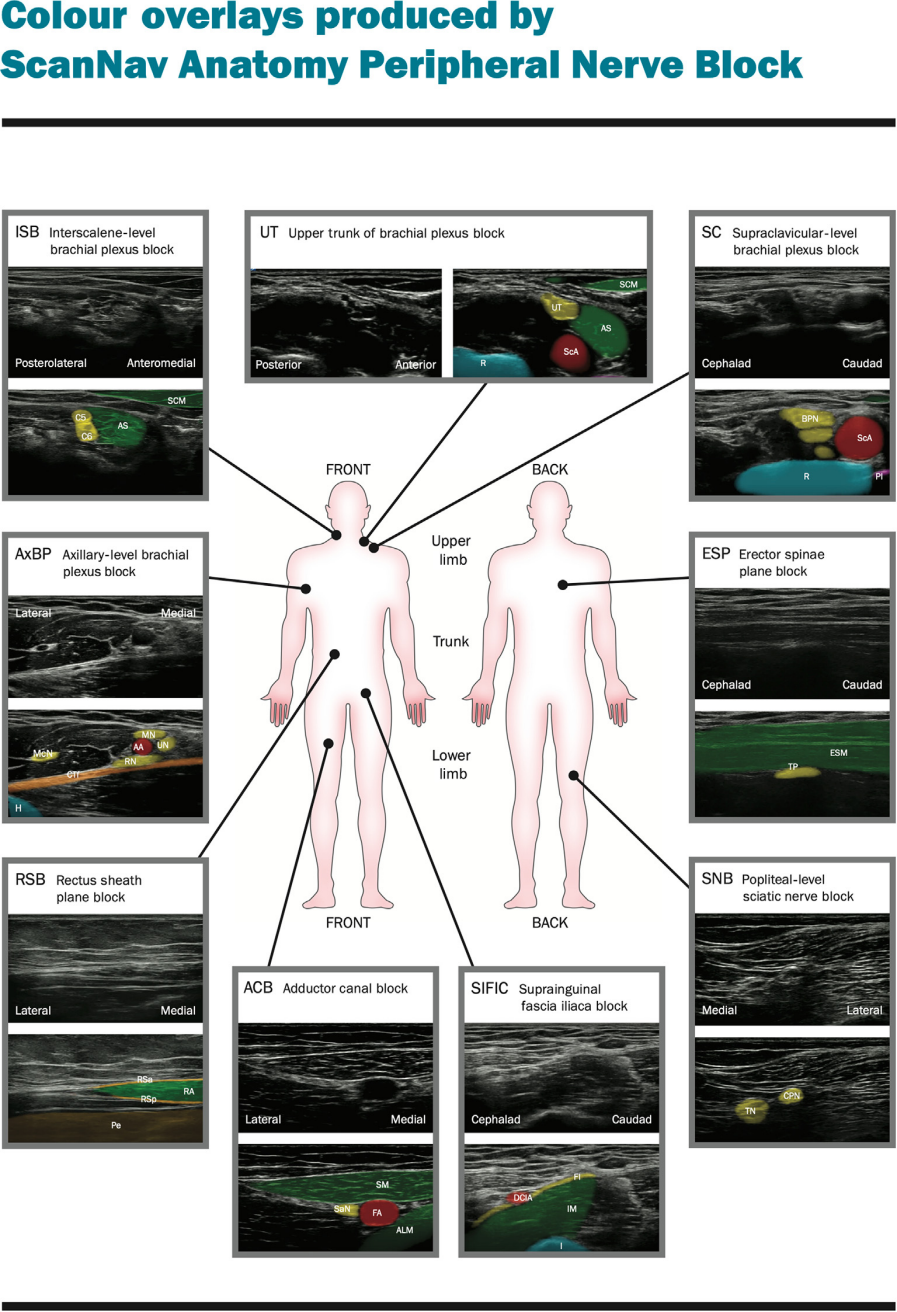
Examples of the artificial-intelligence colour overlay for each peripheral nerve block studied. ALM, adductor longus muscle; AS, anterior scalene; BPN, brachial plexus nerves (trunks/divisions); CPN, common peroneal (fibular) nerve; CTf, fascia overlying conjoint tendon; C5, C5 nerve root; C6, C6 nerve root; DCIA, deep circumflex iliac artery; ESM, erector spinae muscle group (and overlying muscles); FA, femoral artery; FI, fascia iliaca; H, humerus; I, ilium; IM, iliacus/iliopsoas muscle; McN, musculocutaneous nerve; MN, median nerve; Pe, peritoneum and contents; Pl, pleura; R, first rib; RA, rectus abdominis muscle; RN, radial nerve; RSa, anterior layer of rectus sheath; RSp, posterior layer of rectus sheath; SaN, saphenous nerve/nerve complex; ScA, subclavian artery; SCM, sternocleidomastoid muscle; SM, sartorius muscle; TN, tibial nerve; TP, transverse process; UN, ulnar nerve; UT, upper trunk of the brachial plexus.

Table 3 shows a summary of the influence of device highlighting on perceived adverse events according to assessments made by remote experts. Examples of this highlighting are shown in Fig 3 and Supplementary files A–E. Further information is presented in Supplementary file F.

**Table 3:** Influence of highlighting on risk of adverse events and block failure.

Peripheral nerve block	Increase		No change		Reduce		No consensus		Total structures
	%	Structures	%	Structures	%	Structures	%	Structures	
Nerve injury/postoperative neurological symptoms (where nerves highlighted)									
Interscalene-level brachial plexus block	5.0	4	10.0	8	73.8	59	11.2	9	80
Upper-trunk block	0.0	0	62.5	50	37.5	30	0.0	0	80
Supraclavicular-level brachial plexus block	2.5	2	2.5	2	93.8	75	1.2	1	80
Axillary-level brachial plexus block	0.0	0	33.8	27	56.2	45	10.0	8	80
Adductor canal block	0.0	0	72.5	58	21.2	17	6.2	5	80
Popliteal-level sciatic nerve block	2.5	2	0.0	0	95.0	76	2.5	2	80
Average/total	1.7	8	30.2	145	62.9	302	5.2	25	480
Local anaesthetic systemic toxicity (where arteries highlighted)									
Supraclavicular-level brachial plexus block	2.5	2	2.5	2	92.5	74	2.5	2	80
Axillary-level brachial plexus block	2.5	2	1.2	1	91.2	73	5.0	4	80
Suprainguinal fascia iliaca block	0.0	0	8.8	7	81.2	65	10.0	8	80
Adductor canal block	0.0	0	2.5	2	96.2	77	1.2	1	80
Popliteal-level sciatic nerve block	1.2	1	25.0	20	70.0	56	3.8	3	80
Average/total	1.2	5	8.0	32	86.2	345	4.5	18	400
Pneumothorax (only where pleura highlighted)									
Supraclavicular-level brachial plexus block	0.0	0	1.2	1	97.5	78	1.2	1	80
Erector spinae plane block	0.0	0	25.0	20	55.0	44	20.0	16	80
Average/total	0.0	0	13.1	21	76.2	122	10.6	17	160
Peritoneum violation (only where peritoneum highlighted)									
Rectus sheath block	1.2	1	13.8	11	82.5	66	2.5	2	80
Average/total	1.25	1	13.75	11	82.50	66	2.5	2	80
Block failure (all blocks)									
Interscalene-level brachial plexus block	3.8	3	10.0	8	78.8	63	7.5	6	80
Upper-trunk block	2.5	2	8.8	7	81.2	65	7.5	6	80
Supraclavicular-level brachial plexus block	1.2	1	1.2	1	93.8	75	3.8	3	80
Axillary-level brachial plexus block	0.0	0	8.8	7	70.0	56	21.5	17	80
Erector spinae plane block	3.8	3	15.0	12	68.8	55	12.5	10	80
Rectus sheath block	2.5	2	10.0	8	83.8	67	3.8	3	80
Suprainguinal fascia iliaca block	1.2	1	0.0	0	97.5	78	1.2	1	80
Adductor canal block	0.0	0	30.0	24	62.5	50	7.5	6	80
Popliteal-level sciatic nerve block	1.2	1	0.0	0	95.0	76	3.8	3	80
Average/total	1.8	13	9.3	67	81.2	585	7.6	55	720

Nerve highlighting was considered to reduce the risk of nerve injury in 62.9% of cases (302/480; min–max range: 21.2–93.8%), with no change in 30.21% (145/480; 0–72.5%) and an increase in 1.7% (8/480; 0–5.0%). Artery highlighting was considered to reduce the risk of vascular injection in 86.2% (345/400; 70.0–96.25%), with no change in 8.0% (32/400; 1.25–25.0%) and an increase in 1.2% (5/399; 0–2.5%). Pleura highlighting (present in SC and ESP) was considered to reduce the risk of pneumothorax in 76.25% (122/160; 55.0–97.5%), with no change in 13.1% (21/160; 1.25–25.0%), with no reported cases of increased risk. Peritoneum is only visible in the RSB; highlighting was considered to reduce the risk of peritoneum violation in 82.5% (66/80), make no difference in 13.8% (11/80), and increase the risk in 1.2% (1/80).

Highlighting was considered to reduce the risk of block failure in 81.2% (585/720; min–max range: 62.5–97.5%), make no difference in 9.3% (67/720; 0–30.0%), and increase the risk in 1.8% (13/720; 0–3.8%).

## Discussion

This study is reported according to the Consolidated Standards of Reporting Trials-Artificial Intelligence guidelines.^14^ Most prior AI studies of anatomical structure recognition from UGRA images or videos have consisted of data sets from fewer subjects, assessing fewer structures or on fewer videos/images. Of those published, other than this report, only Gungor and colleagues^15^ assessed a commercially available clinical device with clinically relevant endpoints.

We found that ScanNav^TM^ identified anatomical structures essential to safe and efficacious UGRA on real-time ultrasound in 93.5% of cases. The acquisition and interpretation of optimal ultrasound images are fundamental to the practice of UGRA and are a limiting step for non-experts.^3^^,^^11^ Medical image interpretation is known to be fallible and subjective, even amongst experts.^16^, ^17^, ^18^ Data gathered in this study demonstrate the opportunity to augment human interpretation of ultrasound images during UGRA scanning. The structures highlighted by the AI models closely match those that an international consensus of expert opinion recommends that non-experts identify when performing these procedures.^13^

Subjective expert opinion was that highlighting would reduce the risk of recognised complications in 62.9–86.2% of scans. The potential for unintentional needle trauma of a safety critical structure is another limiting factor in the practice of UGRA. Despite the known benefits of UGRA, the majority of patients undergoing surgery amenable to UGRA techniques are not offered a PNB.^6^ Such assistive technology has the potential to reduce complications of UGRA and remove a barrier to clinical practice.

Highlighting by ScanNav in this study was perceived to reduce block failure in 81.2% of scans (according to subjective expert opinion). Ultrasound guidance is associated with improved success rates of PNB, faster onset of sensory block, and reduced incidence of vascular injury and local anaesthetic systemic toxicity.^2^^,^^19^ However, there is still a failure rate to each technique, and the downturn in elective operations conducted during the recent pandemic has led to a commonly held concern over a lack of opportunities to acquire the necessary skills. Medical societies are attempting to promote widespread adoption and standardisation of UGRA.^6^^,^^13^^,^^20^^,^^21^ To support the implementation of these aims, innovation is needed to support clinicians in the delivery of safe and efficacious UGRA.

We show that ScanNav^TM^ holds potential to support ultrasound scanning in UGRA and mitigate the risk of complications or block failure. The device has gained regulatory approval for clinical use in Europe (April 2021), and data from this study contribute to evidence submitted for regulatory review in the USA. This and other similar devices could in time support the widespread practice by non-experts or even novices for ultrasound-guided procedures throughout medicine. For example, emergency-department physicians are often familiar with point-of-care ultrasound and interventional procedures,^22^ and such assistive technology may aid the practice of UGRA in this setting. Its use for painful interventions currently carried out under sedation obviates the risk of airway compromise, reduces the burden of monitoring, and provides a prolonged pain-free period to facilitate hospital discharge or act as a bridge to definitive treatment (e.g. for hip fractures). Beyond UGRA, use of AI in image interpretation has broader implications across medicine and potentially all of ultrasonography,^23^ from screening for developmental dysplasia of the hip^24^ to diagnosis of breast cancer.^25^ The democratisation of ultrasonography will help ensure that patients have access to the most appropriate interventions, supporting the performance of ultrasound-based interventions by non-experts whilst maintaining relevant clinical standards.^26^

The authors recognise limitations to this study. Firstly, our findings must be followed by clinical studies to determine if the predicted benefits are realised in patient outcomes. In particular, use of ultrasound itself has not been shown to reduce the incidence of nerve injury or postoperative neurological symptoms.^2^ Assessing the impact of any ultrasound augmentation technology will require rigorous evaluation. Secondly, the remote expert panels reviewing the videos and highlighting were not present when the subject was scanned. Contemporaneous viewing and interpretation of the ultrasound image provide a richer source of information for the operator, and the expert-panel assessments may have been different with this additional knowledge. However, this limitation is attenuated by the fact that three remote experts assessed each video and could play/pause/review them at any point, changing their assessment if required. Multiple practitioners and the luxury of time or changing clinical opinion are often not afforded to physicians in clinical practice. Thirdly, this study is a subjective assessment of the device according to expert opinion. This is particularly true for findings relating to efficacy and safety. Additional studies are in progress to conduct an objective and pixel-by-pixel assessment of AI highlighting accuracy for structure boundaries (compared with expert interpretation). Whilst this will be useful, it should be noted that such assessments may not always correlate with clinical usefulness, and there is a need for measures of performance beyond accuracy.^27^ The current assessment has been chosen to be consistent with requirements for reporting device performance for regulatory approval published by the US Food and Drug Administration,^28^ which recommends that definitions (e.g. accuracy and FPr/FNr) should be consistent with the intended use of the device. However, it should be noted that there are multiple methods of reporting accuracy of medical devices or tests.^29^ Finally, multiple investigators in this study have been involved with the development and regulatory evaluation of this device. The authors hope that, as the technology becomes more widely available, more anaesthetists will engage in detailed study of this and similar devices to determine their true value to our clinical practice.

Whilst further clinical data on patient outcomes are required to confirm the predicted benefits, these data present the case for the accuracy of ScanNav^TM^ and the potential safety and efficacy benefits in UGRA. This study marks a shift in ultrasonography-guided regional anaesthesia, where technological progress is not restricted to image generation but also to image interpretation.

## Authors' contributions

Study concept/design: JSB, DB-SL, SM, AV, NS

Participant recruitment: JSB, NH, CL, SM, EM, AP, MR, NS, AT, AV, GW

Data collection: JSB, NH, CL, SM, EM, AP, MR, NS, AT, AV, GW

Article preparation: all authors

Article editing: all authors

## Funding

Intelligent Ultrasound Limited (Cardiff, UK) via a grant to JSB administered by the University of Oxford (R70327/CN002).

## Declarations of interest

JSB is a senior clinical advisor for Intelligent Ultrasound, receiving research funding and honoraria. DB-SL is a clinical advisor for Intelligent Ultrasound, receiving honoraria. PAK has acted as a consultant for DeepMind, Roche, Novartis, Apellis, and Bitfount, and he is an equity owner in Big Picture Medical. He has received speaker fees from Heidelberg Engineering, Topcon, Allergan, and Bayer. PAK is supported by a Moorfields Eye Charity Career Development Award (R190028A) and a UK Research and Innovation Future Leaders Fellowship (MR/T019050/1). CL and EM are members of the Executive Board of the European Society of Regional Anaesthesia & Pain Therapy. AP declares honoraria from GE Healthcare, Butterfly Network, Sintetica UK Ltd, and Pacira, and he is the immediate past president of Regional Anaesthesia UK. MR is on the Board of Directors of the American Society of Regional Anesthesia and Pain Medicine. NS is the chief technical officer of Intelligent Ultrasound. AT has received honoraria from Intelligent Ultrasound. JAN is a senior scientific advisor for Intelligent Ultrasound.
