# Supplementary material for: Assistive artificial intelligence for ultrasound image interpretation in regional anaesthesia: an external validation study
Source: Br J Anaesth. 2022 Aug 18;130(2):217–25. doi: 10.1016/j.bja.2022.06.031 (PMC9900723; doi:10.1016/j.bja.2022.06.031)
Supplement: Multimedia component 6 [file mmc6.docx]

**SUPPLEMENTARY FILE F**

**Assistive Artificial Intelligence for Ultrasound Image Interpretation in Regional Anaesthesia:**

**An External Validation Study**

**Authors**

James Bowness^1,2^, David Burckett-St Laurent^3^, Nadia Hernandez^4^, Pearse Keane^5,6^, Clara Lobo^7^, Steve Margetts^8^, Eleni Moka^9^, Amit Pawa^10^, Meg Rosenblatt^11^, Nick Sleep^8^, Alasdair Taylor^12^, Glenn Woodworth^13^, Asta Vasalauskaite^8^, J Alison Noble^14^, Helen Higham^1,15^

**Author Institutions**

1. Oxford Simulation, Teaching and Research Centre, University of Oxford, Oxford, UK
2. Department of Anaesthesia, Aneurin Bevan University Health Board, Newport, UK
3. Department of Anaesthesia, Royal Cornwall Hospitals NHS Trust, Truro, UK
4. Department of Anesthesiology, Memorial Hermann Hospital, Texas Medical Centre, Texas, USA
5. Institute of Ophthalmology, Faculty of Brain Sciences, University College London, London, UK
6. NIHR Biomedical Research Centre, Moorfields Eye Hospital NHS Foundation Trust, London, UK
7. Department of Anesthesiology, Hospital das Forcas Armadas Polo do Porto, Porta, Portugal
8. Intelligent Ultrasound, Cardiff, UK
9. Department of Anaesthesiology, Hellenic Healthcare Group (HHG), Heraklion, Crete, Greece
10. Department of Anaesthesia, Guy’s & St Thomas’ Hospitals NHS Trust, London, UK
11. Mount Sinai Health System, New York, New York, USA
12. Department of Anaesthesia, NHS Tayside, Dundee, UK
13. Department of Anesthesiology & Perioperative Medicine, Oregon Health & Science University, Portland, OR, USA
14. Institute of Biomedical Engineering, University of Oxford, UK
15. Department of Anaesthesia, Oxford University Hospitals NHS Foundation Trust, Oxford, UK

# AI Model Training

Supplementary figure on training data for the ScanNav Anatomy PNB AI models used in this study.


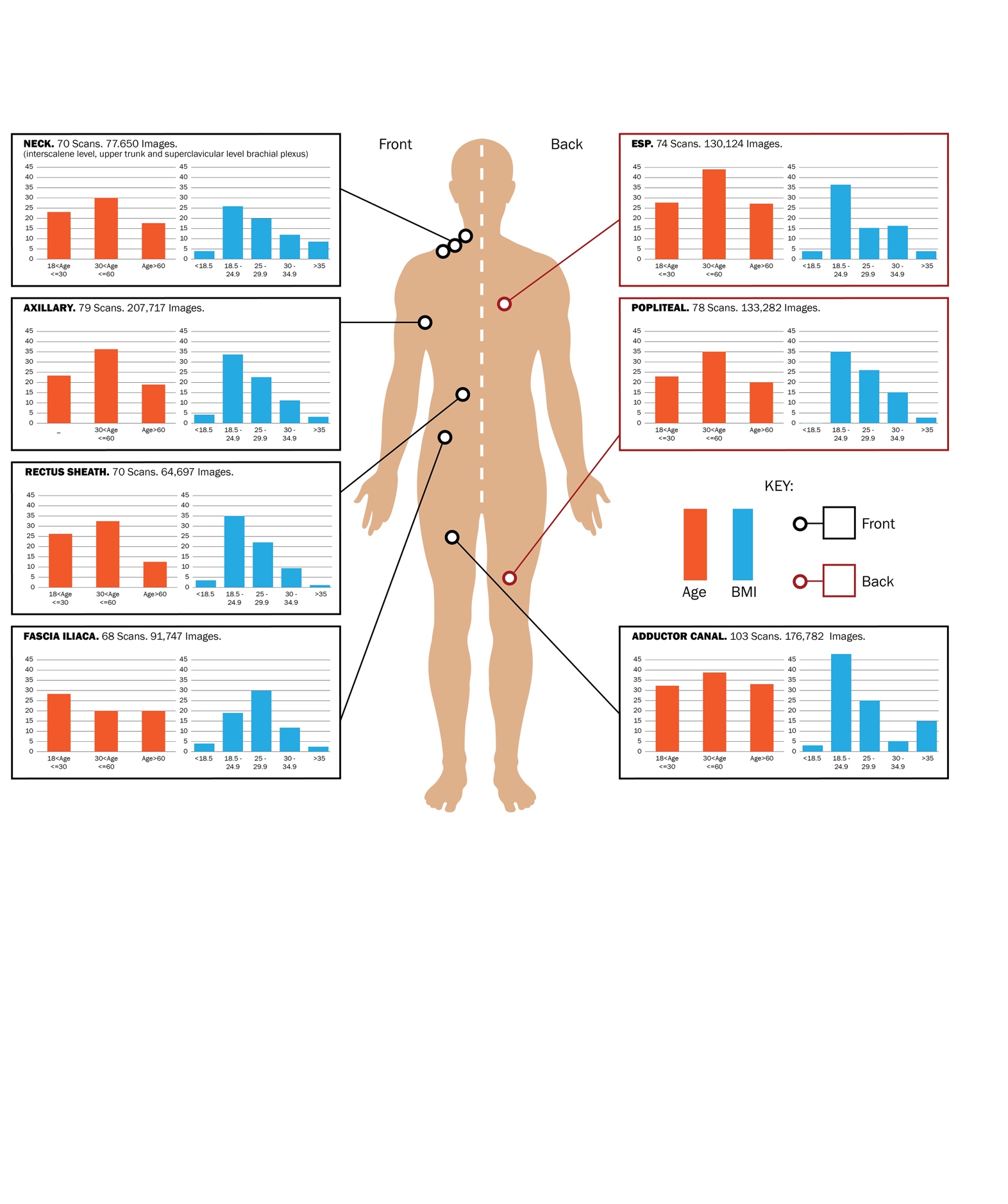


# Block evaluation results

## Summary

**Ax/AxBP** axillary level brachial plexus; **ESP** erector spinae plane; **Is/ISB** interscalene level brachial plexus; **Pop/P-SNB** popliteal level sciatic nerve; **RS/RSB** rectus sheath; **Add/ACB** adductor canal; **UT/ST** upper trunk; **SC** supraclavicular level brachial plexus; **SIFC/SIFIB** suprainguinal fascia iliaca

| Block | Accuracy (95% CI) | Error rate (95% CI) |
| --- | --- | --- |
| AxBP | 0.977 [0.956-0.989] | 0.023 [0.011-0.044] |
| ESP | 0.888 [0.827-0.934] | 0.112 [0.067-0.173] |
| ISB | 0.941 [0.891-0.973] | 0.059 [0.027-0.109] |
| P-SNB | 0.981 [0.946-0.996] | 0.019 [0.004-0.054] |
| RSB | 0.968 [0.926-0.989] | 0.032 [0.011-0.074] |
| ACB | 0.904 [0.946-0.945] | 0.096 [0.055-0.154] |
| UT | 0.909 [0.822-0.963] | 0.091 [0.037-0.178] |
| SC | 0.983 [0.957-0.995] | 0.017 [0.005-0.043] |
| SIFIC | 0.762 [0.686-0.827] | 0.238 [0.173-0.315] |

## Axillary level brachial plexus

### Performance of highlighting of anatomical structures

|  | **Artery** | **Radial Nerve** | **Ulnar Nerve** | **Median Nerve** | **Mc Nerve** | **Total** |
| --- | --- | --- | --- | --- | --- | --- |
| **TP** | 1.0  (80) | 1.0  (77) | 1.0  (80) | 1.0  (79) | 0.725  (50) | **366** |
| **TN** | 0 | 0 | 0 | 0 | 0.145  (10) | **10** |
| **FP** | 0 | 0 | 0 | 0 | 0.014  (1) | **1** |
| **FN** | 0 | 0 | 0 | 0 | 0.116  (8) | **8** |
| ***No consensus**** | *0* | *3* | *0* | *1* | *11* | ***15*** |
| **Total** | **80** | **80** | **80** | **80** | **80** | **400** |

|  | **Positive** | **Negative** | **Total** |
| --- | --- | --- | --- |
| **Positive** | TP  0.951  (366) | FN  0.021  (8) | 374 |
| **Negative** | FP  0.003  (1) | TN  0.026  (10) | 11 |
| **Total** | 367 | 18 | 385 |

| outcome | Rate |
| --- | --- |
| Accuracy (TP + TN) | 0.977 |
| Misidentification (FP) | 0.003 |
| Non-identification (FN) | 0.021 |

### Effect of highlighting on risk of adverse events

|  | **Artery** | **Radial Nerve** | **Ulnar Nerve** | **Median Nerve** | **Mn Nerve** | **Total** | **Percent** |
| --- | --- | --- | --- | --- | --- | --- | --- |
| **Increases** | - | - | - | 1 | - | **1** | **0.25%** |
| **No change** | 1 | 5 | 7 | 7 | 29 | **49** | **12.25%** |
| **Reduces** | 79 | 65 | 69 | 63 | 46 | **322** | **80.50%** |
| *No consensus* | - | 10 | 4 | 9 | 5 | **28** | **7%** |
| **Total** | **80** | **80** | **80** | **80** | **80** | **400** |  |

### Effect of highlighting on risk of PONS

| change in risk | Total | Percent |
| --- | --- | --- |
| Increases | - | 0% |
| No change | 27 | 33.75% |
| Reduces | 45 | 56.25% |
| No consensus | 8 | 10% |
| Total | **80** |  |

### Effect of highlighting on risk of LAST

| change in risk | Total | Percent |
| --- | --- | --- |
| Increases | 2 | 2.5% |
| No change | 1 | 1.25% |
| Reduces | 73 | 91.25% |
| No consensus | 4 | 5% |
| Total | **80** |  |

### Effect of highlighting on risk of Block Failure

| change in risk | Total | Percent |
| --- | --- | --- |
| Increases | - | 0% |
| No change | 7 | 8.75% |
| Reduces | 56 | 70% |
| No consensus | 17 | 21.25% |
| Total | **80** |  |

## Erector Spinae Plane

### Performance of highlighting of anatomical structures

|  | **Transverse process** | **Pleura** | **Total** |
| --- | --- | --- | --- |
| **TP** | 0.707  (53) | 0.571  (44) | **97** |
| **TN** | 0.173  (13) | 0.325  (25) | **38** |
| **FP** | 0 | 0 | **0** |
| **FN** | 0.120  (9) | 0.104  (8) | **17** |
| *No consensus** | *5* | *3* | ***8*** |
| **Total** | **80** | **80** | **160** |

|  | **Positive** | **Negative** | **Total** |
| --- | --- | --- | --- |
| **Positive** | TP  0.638  (97) | FN  0.112  (17) | 114 |
| **Negative** | FP  0  (0) | TN  0.250  (38) | 38 |
| **Total** | 97 | 55 | 152 |

| outcome | Rate |
| --- | --- |
| Accuracy (TP + TN) | 0.888 |
| Misidentification (FP) | 0 |
| Non-identification (FN) | 0.112 |

### Effect of highlighting on risk of adverse events

|  | **Transverse Process** | **Pleura** | **Total** | **Percent** |
| --- | --- | --- | --- | --- |
| **Increases** | 1 | - | **1** | **0.63%** |
| **No change** | 16 | 32 | **48** | **30%** |
| **Reduces** | 53 | 41 | **94** | **58.75%** |
| *No consensus* | 10 | 7 | **17** | **10.63%** |
| **Total** | **80** | **80** | **160** |  |

### Effect of highlighting on risk of Pneumothorax

| change in risk | Total | Percent |
| --- | --- | --- |
| Increases | - | 0% |
| No change | 20 | 25% |
| Reduces | 44 | 55% |
| No consensus | 16 | 20% |
| Total | **80** |  |

### Effect of highlighting on risk of Block Failure

| change in risk | Total | Percent |
| --- | --- | --- |
| Increases | 3 | 3.75% |
| No change | 12 | 15% |
| Reduces | 55 | 68.75% |
| No consensus | 10 | 12.5% |
| Total | **80** |  |

## Interscalene level brachial plexus

### Performance of highlighting of anatomical structures

|  | **C5 Nerve** | **C6 Nerve** | **Total** |
| --- | --- | --- | --- |
| **TP** | 0.92  (69) | 0.897  (70) | **139** |
| **TN** | 0.027  (2) | 0.038  (3) | **5** |
| **FP** | 0 | 0.026  (2) | **2** |
| **FN** | 0.053  (4) | 0.038  (3) | **7** |
| *No consensus** | *5* | *2* | ***7*** |
| **Total** | **80** | **80** | **160** |

|  | **Positive** | **Negative** | **Total** |
| --- | --- | --- | --- |
| **Positive** | TP  0.908  (139) | FN  0.046  (7) | 146 |
| **Negative** | FP  0.013  (2) | TN  0.033  (5) | 7 |
| **Total** | 141 | 12 | 153 |

| outcome | Rate |
| --- | --- |
| Accuracy (TP + TN) | 0.941 |
| Misidentification (FP) | 0.013 |
| Non-identification (FN) | 0.046 |

### Effect of highlighting on risk of adverse events

|  | **C5 Nerve** | **C6 Nerve** | **Total** | **Percent** |
| --- | --- | --- | --- | --- |
| **Increases** | 6 | 2 | **8** | **5%** |
| **No change** | 7 | 9 | **16** | **10%** |
| **Reduces** | 65 | 66 | **131** | **81.875%** |
| *No consensus* | 2 | 3 | **5** | **3.125%** |
| **Total** | **80** | **80** | **160** |  |

### Effect of highlighting on risk of PONS

| change in risk | Total | Percent |
| --- | --- | --- |
| Increases | 4 | 5% |
| No change | 8 | 10% |
| Reduces | 59 | 73.75% |
| No consensus | 9 | 11.25% |
| Total | **80** |  |

### Effect of highlighting on risk of Block Failure

| change in risk | Total | Percent |
| --- | --- | --- |
| Increases | 3 | 3.75% |
| No change | 8 | 10% |
| Reduces | 63 | 78.75% |
| No consensus | 6 | 7.5% |
| Total | **80** |  |

## Popliteal level sciatic nerve

### Performance of highlighting of anatomical structures

|  | **Artery** | **Nerve** | **Total** |
| --- | --- | --- | --- |
| **TP** | 0.7625  (61) | 0.9875  (79) | **140** |
| **TN** | 0.2125  (17) | 0 | **17** |
| **FP** | 0 | 0.0125  (1) | **1** |
| **FN** | 0.025  (2) | 0 | **2** |
| *No consensus** | *0* | *0* | ***0*** |
| **Total** | **80** | **80** | **160** |

|  | **Positive** | **Negative** | **Total** |
| --- | --- | --- | --- |
| **Positive** | TP  0.875  (140) | FN  0.013  (2) | 142 |
| **Negative** | FP  0.006  (1) | TN  0.106  (17) | 18 |
| **Total** | 141 | 19 | 160 |

| outcome | Rate |
| --- | --- |
| Accuracy (TP + TN) | 0.981 |
| Misidentification (FP) | 0.006 |
| Non-identification (FN) | 0.013 |

### Effect of highlighting on risk of adverse events

|  | **Artery** | **Nerve** | **Total** | **Percent** |
| --- | --- | --- | --- | --- |
| **Increases** | 2 | 2 | **4** | **2.5%** |
| **No change** | 25 | - | **25** | **15.6%** |
| **Reduces** | 52 | 76 | **128** | **80%** |
| *No consensus* | 1 | 2 | **3** | **1.87%** |
| **Total** | **80** | **80** | **160** |  |

### Effect of highlighting on risk of PONS

| change in risk | Total | Percent |
| --- | --- | --- |
| Increases | 2 | 2.5% |
| No change | - | 0% |
| Reduces | 76 | 95% |
| No consensus | 2 | 2.5% |
| Total | **80** |  |

### Effect of highlighting on risk of LAST

| change in risk | Total | Percent |
| --- | --- | --- |
| Increases | 1 | 1.25% |
| No change | 20 | 25% |
| Reduces | 56 | 70% |
| No consensus | 3 | 3.75% |
| Total | **80** |  |

### Effect of highlighting on risk of Block Failure

| change in risk | Total | Percent |
| --- | --- | --- |
| Increases | 1 | 1.25% |
| No change | - | 0% |
| Reduces | 76 | 95% |
| No consensus | 3 | 3.75% |
| Total | **80** |  |

## Rectus Sheath

### Performance of highlighting of anatomical structures

|  | **Fascia** | **Peritoneum** | **Total** |
| --- | --- | --- | --- |
| **TP** | 0.99  (75) | 0.97  (74) | **149** |
| **TN** | 0 | 0 | **0** |
| **FP** | 0.01  (1) | 0.05  (4) | **5** |
| **FN** | 0 | 0 | **0** |
| *No consensus** | *4* | *2* | ***6*** |
| **Total** | **80** | **80** | **160** |

|  | **Positive** | **Negative** | **Total** |
| --- | --- | --- | --- |
| **Positive** | TP  0.968  (149) | FN  0 | 149 |
| **Negative** | FP  0.032  (5) | TN  0 | 5 |
| **Total** | 154 | 0 | 154 |

| outcome | Rate |
| --- | --- |
| Accuracy (TP + TN) | 0.968 |
| Misidentification (FP) | 0.032 |
| Non-identification (FN) | 0 |

### Effect of highlighting on risk of adverse events

|  | **Fascia** | **Peritoneum** | **Total** | **Percent** |
| --- | --- | --- | --- | --- |
| **Increases** | 1 | 1 | **2** | **1.25%** |
| **No change** | 4 | 13 | **17** | **10.625%** |
| **Reduces** | 69 | 65 | **134** | **83.75%** |
| *No consensus* | 6 | 1 | **7** | **4.375%** |
| **Total** | **80** | **80** | **160** |  |

### Effect of highlighting on risk of Peritoneum Violation

| change in risk | Total | Percent |
| --- | --- | --- |
| Increases | 1 | 1.25% |
| No change | 11 | 13.75% |
| Reduces | 66 | 82.5% |
| No consensus | 2 | 2.5% |
| Total | **80** |  |

### Effect of highlighting on risk of Block Failure

| change in risk | Total | Percent |
| --- | --- | --- |
| Increases | 2 | 2.5% |
| No change | 8 | 10% |
| Reduces | 67 | 83.75% |
| No consensus | 3 | 3.75% |
| Total | **80** |  |

## Adductor canal

### Performance of highlighting of anatomical structures

|  | **Artery** | **Nerve** | **Total** |
| --- | --- | --- | --- |
| **TP** | 1.0  (79) | 0.74  (57) | **136** |
| **TN** | 0 | 0.065  (5) | **5** |
| **FP** | 0 | 0 | **0** |
| **FN** | 0 | 0.195  (15) | **15** |
| *No consensus** | *1* | *3* | ***4*** |
| **Total** | **80** | **80** | **160** |

|  | **Positive** | **Negative** | **Total** |
| --- | --- | --- | --- |
| **Positive** | TP  0.872  (136) | FN  0.096  (15) | 151 |
| **Negative** | FP  0 | TN  0.032  (5) | 5 |
| **Total** | 136 | 20 | 156 |

| outcome | Rate |
| --- | --- |
| Accuracy (TP + TN) | 0.904 |
| Misidentification (FP) | 0 |
| Non-identification (FN) | 0.096 |

### Effect of highlighting on risk of adverse events

|  | **Artery** | **Nerve** | **Total** | **Percent** |
| --- | --- | --- | --- | --- |
| **Increases** | 0 | 0 | **0** | **0%** |
| **No change** | 2 | 27 | **29** | **18.125%** |
| **Reduces** | 76 | 48 | **124** | **77.5%** |
| *No consensus* | 2 | 5 | **7** | **4.375%** |
| **Total** | **80** | **80** | **160** |  |

### Effect of highlighting on risk of PONS

| change in risk | Total | Percent |
| --- | --- | --- |
| Increases | - | 0% |
| No change | 58 | 72.5% |
| Reduces | 17 | 21.25% |
| No consensus | 5 | 6.25% |
| Total | **80** |  |

### Effect of highlighting on risk of LAST

| change in risk | Total | Percent |
| --- | --- | --- |
| Increases | - | 0% |
| No change | 2 | 2.5% |
| Reduces | 77 | 96.25% |
| No consensus | 1 | 1.25% |
| Total | **80** |  |

### Effect of highlighting on risk of Block Failure

| change in risk | Total | Percent |
| --- | --- | --- |
| Increases | - | 0% |
| No change | 24 | 30% |
| Reduces | 50 | 62.5% |
| No consensus | 6 | 7.5% |
| Total | **80** |  |

## Upper Trunk of Brachial Plexus

### Performance of highlighting of anatomical structures

|  | **Nerve** |
| --- | --- |
| **TP** | 69 |
| **TN** | 1 |
| **FP** | 4 |
| **FN** | 3 |
| *No consensus** | *3* |
| **Total** | **80** |

|  | **Positive** | **Negative** | **Total** |
| --- | --- | --- | --- |
| **Positive** | TP  0.896  (69) | FN  0.039  (3) | 72 |
| **Negative** | FP  0.052  (4) | TN  0.013  (1) | 5 |
| **Total** | 73 | 4 | 77 |

| outcome | Rate |
| --- | --- |
| Accuracy (TP + TN) | 0.909 |
| Misidentification (FP) | 0.052 |
| Non-identification (FN) | 0.039 |

### Effect of highlighting on risk of adverse events

|  | **Nerve** | **Percent** |
| --- | --- | --- |
| **Increases** | 2 | **2.5%** |
| **No change** | 8 | **10%** |
| **Reduces** | 63 | **78.75%** |
| *No consensus* | 7 | **8.75%** |
| **Total** | **80** |  |

### Effect of highlighting on PONS

| change in risk | Total | Percent |
| --- | --- | --- |
| Increases | - | 0% |
| No change | 50 | 62.5% |
| Reduces | 30 | 37.5% |
| No consensus | - | 0% |
| Total | **80** |  |

### Effect of highlighting on Block Failure

| change in risk | Total | Percent |
| --- | --- | --- |
| Increases | 2 | 2.5% |
| No change | 7 | 8.75% |
| Reduces | 65 | 81.25% |
| No consensus | 6 | 7.5% |
| Total | **80** |  |

## Supraclavicular level brachial plexus

### Performance of highlighting of anatomical structures

|  | **Artery** | **Nerve** | **Pleura** | **Total** |
| --- | --- | --- | --- | --- |
| **TP** | 0.95  (75) | 0.99  (78) | 0.94  (73) | **226** |
| **TN** | 0.025  (2) | 0 | 0.051  (4) | **6** |
| **FP** | 0.013  (1) | 0 | 0.013  (1) | **2** |
| **FN** | 0.013  (1) | 0.013  (1) | 0 | **2** |
| *No consensus** | *1* | *1* | *2* | **4** |
| **Total** | **80** | **80** | **80** | **240** |

|  | **Positive** | **Negative** | **Total** |
| --- | --- | --- | --- |
| **Positive** | TP  0.958  (226) | FN  0.008  (2) | 228 |
| **Negative** | FP  0.008  (2) | TN  0.025  (6) | 8 |
| **Total** | 228 | 8 | 236 |

| outcome | Rate |
| --- | --- |
| Accuracy (TP + TN) | 0.983 |
| Misidentification (FP) | 0.008 |
| Non-identification (FN) | 0.008 |

### Effect of highlighting on risk of adverse events

|  | **Artery** | **Nerve** | **Pleura** | **Percent** |
| --- | --- | --- | --- | --- |
| **Increases** | 1 | 1 | - | **0.83%** |
| **No change** | 2 | 1 | 3 | **2.5%** |
| **Reduces** | 75 | 75 | 74 | **93.33%** |
| *No consensus* | 2 | 3 | 3 | **3.33%** |
| **Total** | **80** | **80** | **80** |  |

### Effect of highlighting on risk of PONS

| change in risk | Total | Percent |
| --- | --- | --- |
| Increases | 2 | 2.5% |
| No change | 2 | 2.5% |
| Reduces | 75 | 93.75% |
| No consensus | 1 | 1.25% |
| Total | **80** |  |

### Effect of highlighting on risk of Pneumothorax

| change in risk | Total | Percent |
| --- | --- | --- |
| Increases | - | 0% |
| No change | 1 | 1.25% |
| Reduces | 78 | 97.5% |
| No consensus | 1 | 1.25% |
| Total | **80** |  |

### Effect of highlighting on risk of LAST

| change in risk | Total | Percent |
| --- | --- | --- |
| Increases | 2 | 2.5% |
| Reduces | 74 | 92.5% |
| No change | 2 | 2.5% |
| No consensus | 2 | 2.5% |
| Total | **80** |  |

### Effect of highlighting on Block Failure

| change in risk | Total | Percent |
| --- | --- | --- |
| Increases | 1 | 1.25% |
| No change | 1 | 1.25% |
| Reduces | 75 | 93.75% |
| No consensus | 3 | 3.75% |
| Total | **80** |  |

## Suprainguinal fascia iliaca plane

### Performance of highlighting of anatomical structures

|  | **Artery** | **Fascia** | **Total** |
| --- | --- | --- | --- |
| **TP** | 0.41  (29) | 0.96  (77) | **106** |
| **TN** | 0.11  (8) | 0.0125  (1) | **9** |
| **FP** | 0.44  (31) | 0.025  (2) | **33** |
| **FN** | 0.04  (3) | 0 | **3** |
| *No consensus** | *9* | *0* | ***9*** |
| **Total** | **80** | **80** | **160** |

|  | **Positive** | **Negative** | **Total** |
| --- | --- | --- | --- |
| **Positive** | TP  0.702  (106) | FN  0.02  (3) | 109 |
| **Negative** | FP  0.219  (33) | TN  0.06  (9) | 42 |
| **Total** | 139 | 12 | 151 |

| outcome | Rate |
| --- | --- |
| Accuracy (TP + TN) | 0.762 |
| Misidentification (FP) | 0.219 |
| Non-identification (FN) | 0.02 |

### Effect of highlighting on risk of adverse events

|  | **Artery** | **Fascia** | **Total** | **Percent** |
| --- | --- | --- | --- | --- |
| **Increases** | - | - | **0** | **0%** |
| **No change** | 25 | 1 | **26** | **16.25%** |
| **Reduces** | 41 | 76 | **117** | **73.1%** |
| *No consensus* | 14 | 3 | **17** | **10.625%** |
| **Total** | **80** | **80** | **160** |  |

### Effect of highlighting on risk of LAST

| change in risk | Total | Percent |
| --- | --- | --- |
| Increases | - | 0% |
| No change | 7 | 8.75% |
| Reduces | 65 | 81.25% |
| No consensus | 8 | 10% |
| Total | **80** |  |

### Effect of highlighting on risk of Block Failure

| change in risk | Total | Percent |
| --- | --- | --- |
| Increases | 1 | 1.25% |
| No change | - | 0% |
| Reduces | 78 | 97.5% |
| No consensus | 1 | 1.25% |
| Total | **80** |  |
